# Sensitive, non-invasive detection of chronic wasting disease in wild and captive white-tailed deer using fecal volatile profiling

**DOI:** 10.1128/msphere.00351-25

**Published:** 2025-08-18

**Authors:** Amalia Z. Berna, Tzvi Y. Pollock, Yang Liu, Michelle Gibison, Amritha Mallikarjun, Joey Logan, Cynthia M. Otto, Audrey R. Odom John

**Affiliations:** 1 Division of Infectious Diseases, Children’s Hospital of Philadelphia, Philadelphia, Pennsylvania, USA; 2 School of Veterinary Medicine, Wildlife Futures Program, New Bolton Center, University of Pennsylvania 6572, Philadelphia, Pennsylvania, USA; 3 School of Veterinary Medicine, Penn Vet Working Dog Center, University of Pennsylvania 6572, Philadelphia, Pennsylvania, USA; 4 Department of Biomedical and Health Informatics, Children’s Hospital of Philadelphia, Philadelphia, Pennsylvania, USA; 5 School of Veterinary Medicine, Department of Clinical Sciences and Advanced Medicine, University of Pennsylvania 6572, Philadelphia, Pennsylvania, USA; 6 Perelman School of Medicine, University of Pennsylvania 6572, Philadelphia, Pennsylvania, USA; University of Michigan Medical School, Ann Arbor, Michigan, USA

**Keywords:** prions, volatile organic compounds, chronic wasting disease

## Abstract

Chronic wasting disease (CWD) is a universally fatal, transmissible prion disease affecting cervids. Primarily found among deer populations in North America, the disease has spread across the continent and made forays into Europe and Asia as well. Currently, accurate methods for detecting CWD infection require postmortem dissection of the lymph nodes and brainstem of affected animals. New, high-sensitivity methods of identifying CWD in living animals are sorely needed to help curb the spread of this devastating disease in captive and wild deer. Here, we use two-dimensional gas chromatography-mass spectrometry (GCxGC-MS) to detect volatile organic compounds (VOCs) released from feces of white-tailed deer (WTD) for differentiation of the feces of CWD-negative and CWD-positive animals. We report 4 discrete VOCs in captive WTD and 10 discrete VOCs in wild WTD, with which we can discriminate CWD-positive and CWD-negative samples. Additionally, we evaluate the ability to detect biomarkers associated with CWD early during disease progression, by comparing samples from the early stage of infection with samples from the late stage and uninfected WTD. Our data suggest that detection of VOCs from the feces of WTD—both in captive and wild populations—can serve as a highly sensitive and non-invasive technique for identifying CWD infection in living animals.

**IMPORTANCE:**

Chronic wasting disease (CWD) is a deadly, transmissible prion disease of cervids. The spread of CWD is increasing among both wild and captive deer populations; however, the options to detect in living animals are limited. Diagnosing CWD early would allow more effective control over the spread between animals and contamination of the environment. Our research presents a method of determining CWD infection through the detection of disease-associated odor molecules in the feces of affected deer. This methodology lays the foundation for rapid, non-invasive diagnosis of CWD in living white-tailed deer, allowing for the development of tools to enhance control of this devastating disease’s spread among both captive and wild populations.

## INTRODUCTION

Chronic wasting disease (CWD) is a transmissible spongiform encephalopathy of cervids. The etiological agent of CWD is the misfolded form of the prion protein (PrPCWD), which associates with the properly folded form of the protein in the brain and triggers a conformational change (1, 2). CWD is uniformly fatal and can spread rapidly to devastate herds (3). Infectious prions have been found in the peripheral tissues of infected animals, as well as detected in excreta such as feces, urine, saliva, and blood (4–9).

There is no treatment for CWD, and removal from the environment is difficult, as the misfolded protein is resistant to decontamination (10, 11). Additionally, a relatively low titer of PrPCWD appears sufficient for oral transmission, contributing to rapid spread (5). As such, prompt detection is critical to disease control. Unfortunately, the standard method for diagnosis of CWD relies on necropsy and evaluation of the retropharyngeal lymph nodes (RPLNs) and brain stem for PrPCWD aggregates by immunohistochemistry (IHC) or enzyme-linked immunosorbent assay (ELISA) (12). Newer research methods like real-time quaking-induced conversion (RT-QuIC) require samples to be collected via tissue biopsy for best results (4, 13). The lack of non-invasive diagnostic techniques in living animals has proven an obstacle to disease monitoring and control, especially early during disease progression. As CWD continues to spread, the dire need for antemortem and environmental screening is clear.

Recently, volatile organic compounds (VOCs) have been attractive candidates for minimally invasive disease biomarkers. VOCs are produced by all living organisms as communicative signals or metabolic products. The detection of VOCs in human breath, using methods such as gas chromatography/mass spectrometry (GC-MS) or electronic nose systems, has revealed distinct volatiles associated with human diseases such as tuberculosis, malaria, and liver disease (14–18). In livestock, VOC detection in breath and feces has allowed diagnosis of diseases such as bovine tuberculosis (19). A study demonstrated that fecal VOC profiles from certain deer populations can differentiate CWD status (20). This suggests a plausible mechanism by which dogs can differentiate between feces from CWD-positive and CWD-negative deer (21).

In this study, we aimed to use two-dimensional GCxGC-MS VOC detection methods to further characterize the volatile profile of CWD-associated fecal odors, in both wild and captive deer populations. Given the difficulty of diagnosing CWD early during infection, we also sought to evaluate whether VOCs could discriminate the early-stage infection. We find that a small handful of VOCs distinguish CWD-positive and CWD-negative samples with good accuracy, both in wild and captive animals. Additionally, our results support the potential for fecal volatiles to identify early-stage CWD-infected animals in the wild.

## RESULTS

### Four volatile compounds discriminate CWD-positive cervids in captivity

**Fig 1:**
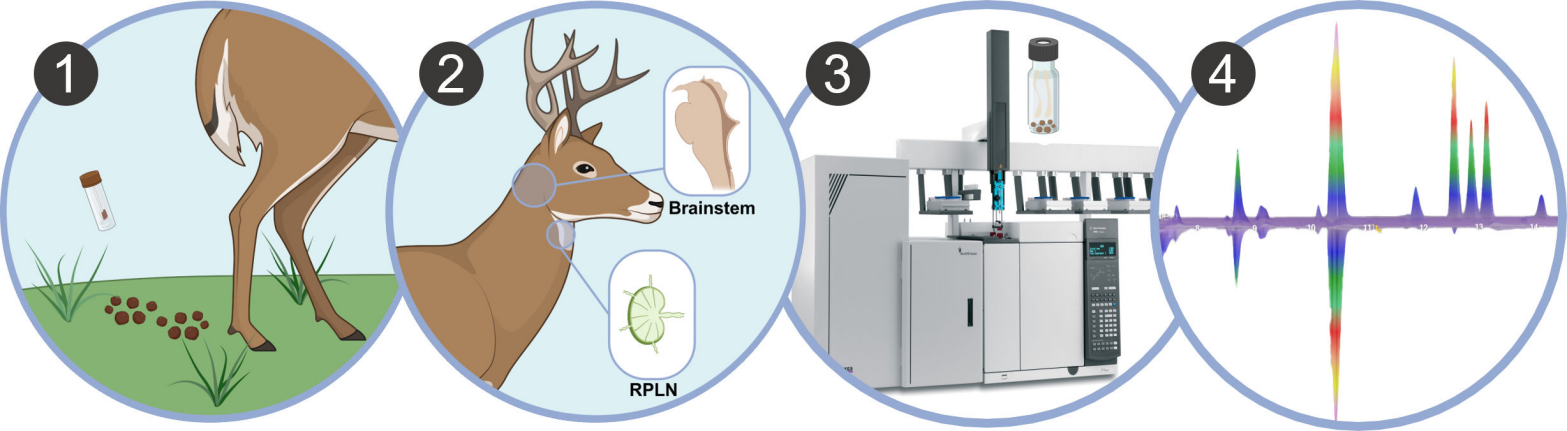
(1) Fecal samples from white-tail deer are tested for CWD-associated volatiles by GCxGC-MS. (2) Fecal samples were removed postmortem from captive or wild WTD. (3) Animals were classified as CWD-positive or CWD-negative by diagnostic IHC of brainstem and retropharyngeal lymph nodes (RPLNs). (4) Headspace solid-phase microextraction and GCxGC-MS analysis were performed on fecal samples. Untargeted data analysis identified volatile compounds from fecal pellets with discriminatory potential between classified deer.

**Fig 2:**
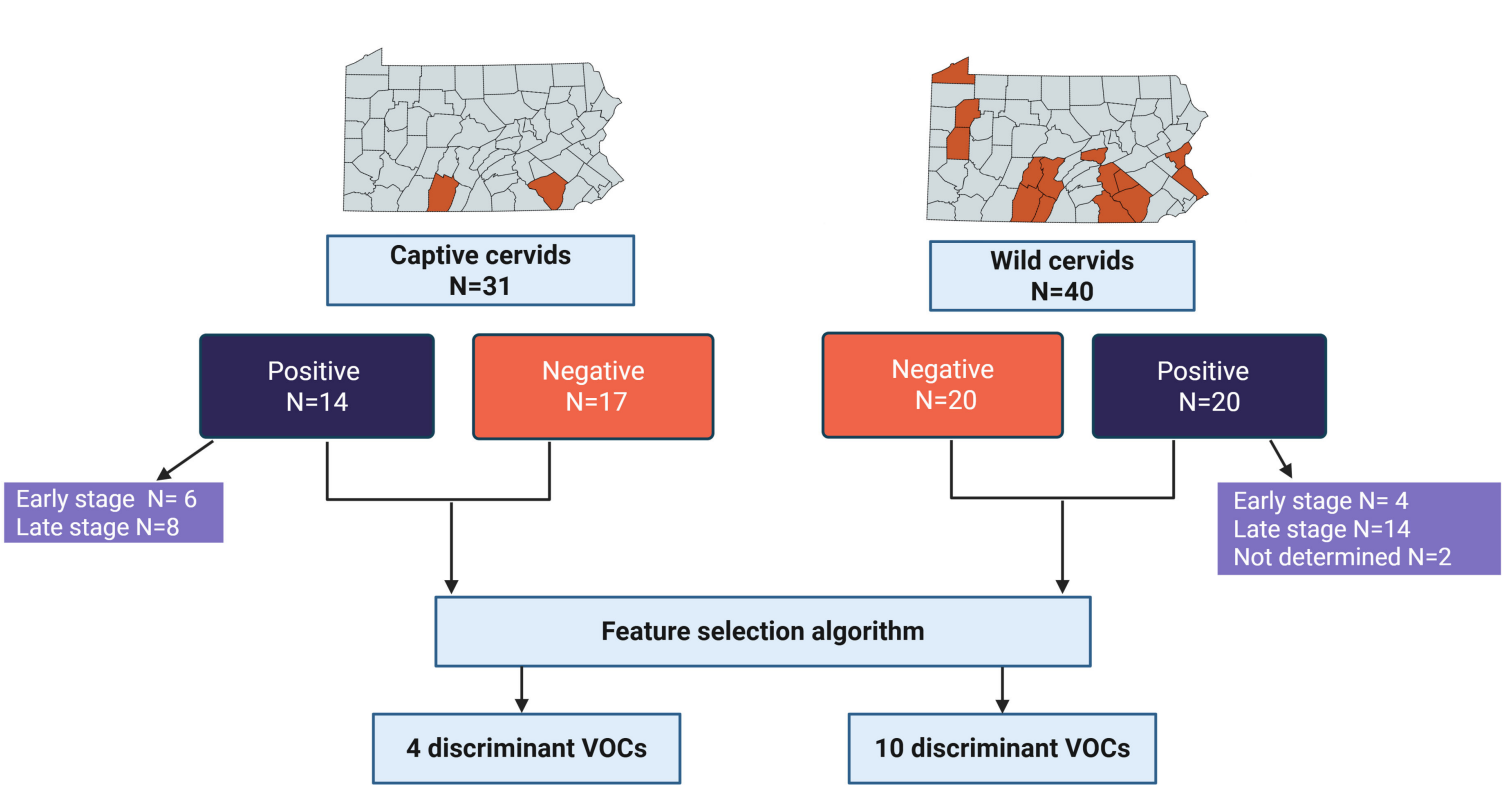
Sources and infection status of animals tested. Captive and wild WTD were sampled from the highlighted counties of Pennsylvania, USA. Animals tested were determined to be CWD-positive or CWD-negative as indicated, and CWD-positive animals were characterized as early-stage when PrPCWD was detected in the RPLN and late-stage CWD when PrPCWD was detected in both RPLN and brainstem. Positive and negative samples were both used in feature selection, which found discriminant VOCs as shown.

A previous study conducted using 1-dimensional GC-MS distinguished feces from CWD-positive and CWD-negative white-tailed deer (WTD) in captive herds using a set of seven VOCs (20). We sought to utilize our two-dimensional GCxGC-MS system to further define any CWD-distinguishing fecal volatiles. Fecal samples were collected postmortem from captive animals confirmed by IHC of the RPLNs and brainstem to be either CWD-positive or CWD-not detected (hereafter referred to as negative) (Fig. 1). VOCs arising from 31 fecal samples (*N* = 14 positive, *N* = 17 negative) were analyzed through solid phase microextraction (SPME) and GCxGC-MS (Fig. 2). Subsequent use of a feature selection algorithm identified four VOCs as discriminant markers of CWD-positivity, shown in Table 1.

**TABLE 1:** Volatile identification of discriminant features found in fecal matter from captive cervids positive and negative for CWD^*a*^

Compound name	Rt1 (min)	Rt2 (s)	*m/z*	Structure
Unknown 1	17.5	1.0	98	
α-Methylstyrene	25.8	0.25	117	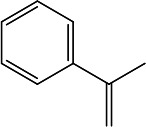
1-Hexanol, 2-ethyl-	33.1	0.25	72	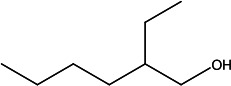
4-Cyanocyclohexene	36.2	0.25	80	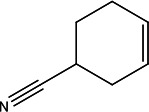

^*a*^ Rt1 and Rt2 indicate the retention time for first dimension column D1 (min) and second dimension column D2 (s), respectively, and extracted *m/z* (mass-to-charge ratio) used for analysis.

**Fig 3:**
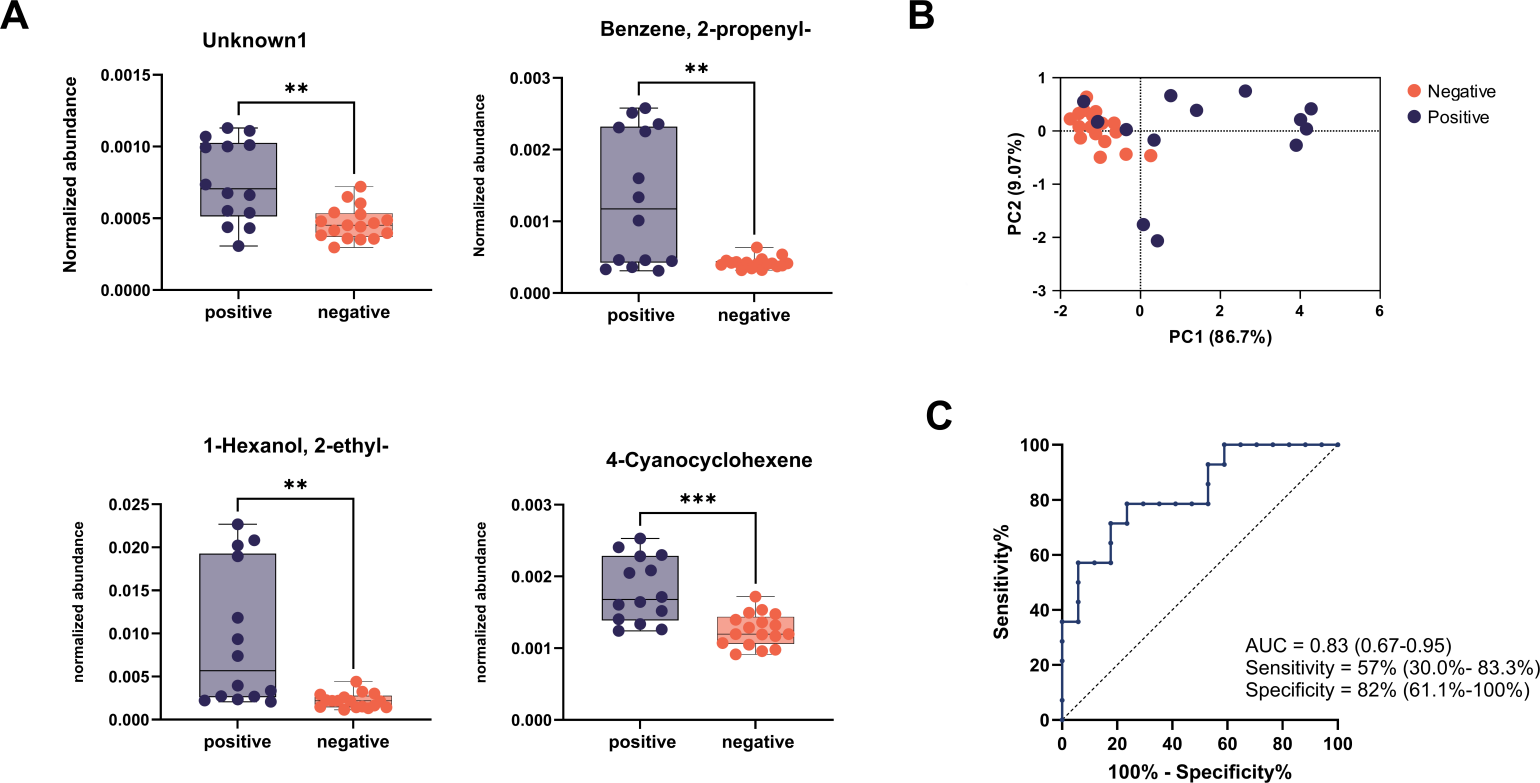
Four VOCs discriminate between CWD-positive and CWD-negative fecal samples from captive WTD. (**A**) Abundances of four volatiles identified during feature selection were compared between positive (*N* = 14) and negative (*N* = 17) samples. Abundances were normalized to internal standard compounds. (**B**) Principal components analysis visualizing the distances between positive and negative fecal sample volatile profiles. (**C**) Receiver operating characteristic (ROC) curve with 95% confidence intervals for AUC, sensitivity, and specificity using the four discriminant volatiles to predict infection status.

Directly comparing levels of these volatiles revealed a significantly greater abundance of all four compounds in CWD-positive cervids (Fig. 3A). Principal component analysis (PCA) used only for visualization purposes revealed distinct clusters of CWD-positive and CWD-negative samples (Fig. 3B). Random forest modeling with Gini importance measurement identified the four VOCs in Table 1 as potentially discriminant volatiles (Fig. S1A), yielding a sensitivity of 57% and a specificity of 82%, for an overall accuracy of 71% (Fig. 3C). One of these discriminant volatiles, 1-hexanol, 2-ethyl-, distinguished CWD infection on its own with a sensitivity of 94% and a specificity of 64% (Fig. S1B). Overall, our model boasted moderate accuracy but high specificity, suggesting fecal VOCs might be best suited for screening purposes (22).

Asymptomatic animals can spread infectious PrPCWD in the environment, and so minimally invasive diagnostic techniques that can detect early-stage infection are required. To this end, we compared early-stage and late-stage CWD. Presence of PrPCWD in the RPLNs alone was defined as early-stage, while presence in both RPLNs and brainstem was defined as late-stage (23). Our preliminary analysis revealed significantly decreased levels of the VOCs 1-hexanol, 2-ethyl- and 2-propenyl-benzene in early-stage, compared to late-stage CWD (Fig. S2A and B). However, no clustering was observed between early-stage CWD-infected captive cervids and healthy controls or early-stage and late-stage CWD among wild cervids by PCA (Fig. S2C and D). Since our sample size was small, this preliminary analysis is underpowered, and future studies will require a larger population of early-stage CWD.

Finally, we manually searched for a subset of the volatiles previously found to be discriminant (20). No significant differences in these compounds were detected between CWD-positive and CWD-negative samples (Fig. S3A).

### Ten volatile compounds discriminate CWD-positive cervids in field settings

VOC-based detection of CWD infection has previously focused on captive WTD; however, CWD infects both captive and wild populations (3). We therefore used our GCxGC-MS system to evaluate whether fecal VOCs might also discriminate between CWD-positive and CWD-negative animals in field settings. Forty fecal samples (*N* = 20 positive and *N* = 20 negative) were procured from wild WTD collected from hunter harvests, roadkill, or clinical suspects (Fig. 2). Animals were confirmed to be CWD-positive or CWD-negative by repeated ELISA and confirmatory IHC (Fig. 1) (12). Fecal samples were subjected to SPME isolation of VOCs, followed by GCxGC-MS analysis. Feature discovery identified 10VOCs as potentially discriminant features of CWD-positivity, shown in Table 2.

**TABLE 2:** Volatile identification of discriminant features found in fecal matter from wild-type cervids positive and negative for CWD^*a*^

Compound number	Compound name	Rt1	Rt2	*m/z*	Structure
1	2-Pentanone	12.0	0.625	43	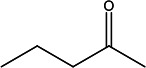
2	2-Pentanone, 4-methyl-	12.6	0.625	100	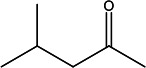
3	2,3-Pentanedione	14.4	0.625	101	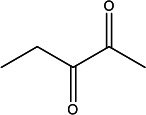
4	2-Hexanone	15.1	0.625	43	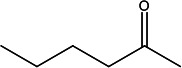
5	2-Butenal, 2-methyl-, (E)-	15.8	1.0	84	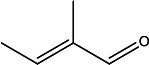
6	Dodecane	19.6	1.0	141	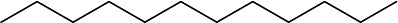
7	Unknown2	29.6	1.0	109	
8	Benzaldehyde	34.5	0.625	75	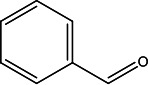
9	*1,3-Benzenediol, 2-methyl-	35.9	0.25	106	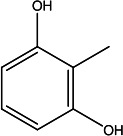
10	*Benzyl alcohol, p-hydroxy-α-[(methylamino)methyl]-	56.03	0.25	167	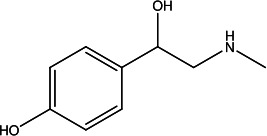

^*a*^ Rt1 and Rt2 indicated the retention time for first dimension column D1 (min) and second dimension column D2 (s), respectively, and extracted mass-to-charge ratio (*m/z*) used for data analysis (* VOCs were tentatively identified with NIST library using >60% match).

**Fig 4:**
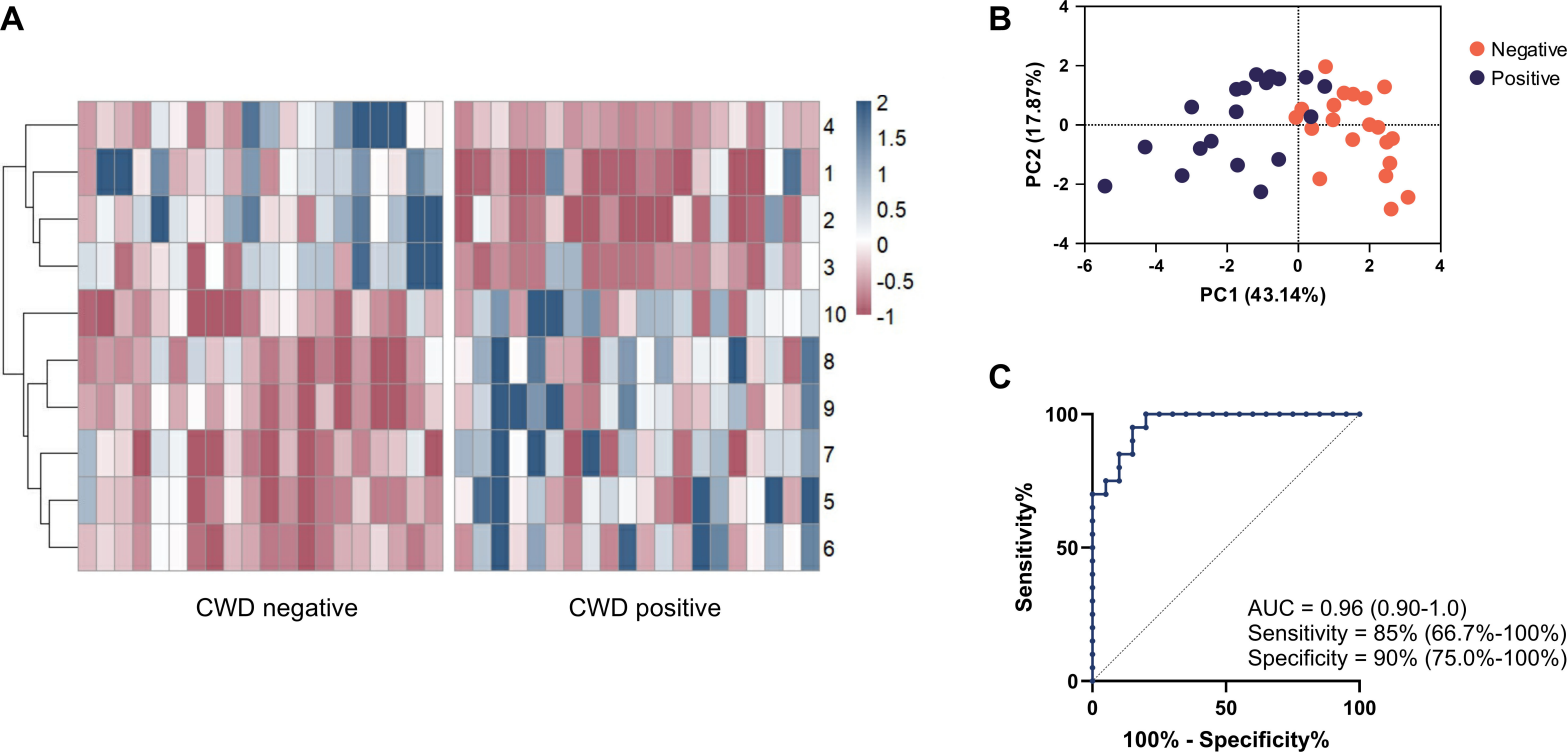
Ten VOCs discriminate between CWD-positive and CWD-negative fecal samples from wild WTD. (**A**) *z* score abundances of ten discriminant volatiles in CWD-negative (*N* = 20) and CWD-positive (*N* = 20) fecal samples. Compound number correlates to the respective compound in Table 2. (**B**) Principal components analysis visualizing the distances between positive and negative fecal sample volatile profiles. (**C**) Receiver operating characteristic (ROC) curve with 95% confidence intervals for AUC, sensitivity, and specificity using the 10 discriminant volatiles to predict infection status.

Four of the compounds isolated in our screen were lower in the feces of CWD-positive animals compared to CWD-negative animals, while the reverse was true of the other six compounds (Fig. 4A). PCA revealed that wild CWD-positive and CWD-negative samples formed two distinct clusters, indicating substantial differences in their VOC profiles (Fig. 4B). Random Forest modeling using these 10 discriminant VOCs resulted in 85% sensitivity and 90% specificity, for an accuracy of 88% (Fig. 4C). These data suggest that fecal volatile profiling has strong potential as a non-invasive, highly accurate tool for biomarker detection of CWD in wild populations.

Much like in captive cervids, early detection of CWD in wild cervid populations is crucial to combatting spread. Our preliminary analysis demonstrated no separation between the VOC profiles of early-stage and late-stage CWD among wild cervids (Fig. S5A). As we observed that levels of 21-hexanol, -ethyl- and 2-propenyl benzene were elevated in late-stage CWD among captive animals (Fig. S2A and B), we compared levels of these two volatiles in wild samples as well. We observed no separation between early-stage and late-stage CWD in wild cervids (Fig. S5B). Furthermore, PCA revealed that early-stage CWD samples cluster away from healthy controls (Fig. S5C). These data suggest that fecal VOC collection can detect even early-stage CWD in wild cervids. As with our data in captive cervids, however, the small sample size of early-stage CWD (*N* = 4) is a major limitation, and further studies are required.

We again searched for a subset of the discriminant volatiles identified in a previous study of captive WTD (20), finding that 1-butanol levels were increased in feces from CWD-negative deer, while 3-methyl-indole levels were increased in feces from CWD-positive deer (Fig. S3B).

## DISCUSSION

The cervid prion disease CWD has no known treatments and is uniformly fatal. A low titer of PrPCWD is sufficient to transmit orally, leading to rapid spread through herds of WTD (5). Furthermore, animals can shed infectious prions long before symptoms (6). The need for new forms of minimally invasive, antemortem testing for CWD is clear and pressing (3). Diagnosis of infection using VOC profiling is a growing field, with studies detecting infections such as tuberculosis and malaria, among others (24, 25). We demonstrate in this study that fecal volatiles differentiate feces from CWD-positive and CWD-negative deer with high accuracy. Our work demonstrates the promise of fecal VOC analysis as a method of CWD surveillance in captive and wild animal populations.

Our study reveals four volatile compounds, identified in Table 1, which discriminated CWD infection in captive WTD with a classification accuracy of 71% (Fig. 3). Likewise, our study reveals 10 volatile compounds, identified in Table 2, which discriminated CWD infection in wild WTD with a classification accuracy of 88% (Fig. 4). Interestingly, many of the VOCs identified in this study have been previously associated with diseases involving gut dysfunction, such as Crohn’s disease, ulcerative colitis, and celiac disease (Table S1 containing full references), and most are of exogenous origin (26, 27). This is particularly relevant given recent findings that CWD is associated with alterations in the gut microbiome of WTD (28). Such dysbiosis may compromise gut barrier integrity, potentially allowing translocation of metabolites into systemic circulation. The VOCs identified in this study did not overlap with those seen previously (20); differences between the volatiles found here and in previous work may derive from the use of higher resolution instrumentation, as well as from natural variation between populations of captive and wild cervids. Additional factors—including animal location, age, and sex—that were not recorded in the early study (20) may also contribute to differences in VOC profiles. Moreover, host genotype polymorphisms, which have been associated with CWD infection status in deer (29), could further influence volatile production. Non-overlapping profiles within our studies and between studies suggest that VOC biomarkers of CWD may be context-dependent and influenced by host factors (e.g., genetics, diet, and microbiota). While this variability presents challenges for biomarker generalizability, it also underscores the importance of validating candidate VOCs across diverse populations and environmental settings. Our work here represents a strong framework for future diagnostic testing, emphasizing the need for broader validation in varied cervid cohorts.

Perhaps surprisingly, the fecal VOCs that proved discriminant for captive WTD and wild WTD did not feature any common compounds. Additionally, fecal VOC profiles of wild WTD demonstrated far greater sensitivity than captive WTD, with 85% sensitivity for the former and 57% sensitivity for the latter (Fig. 3C and 4C). One possible cause for these discrepancies lies in the effect of captivity on the intestinal microbiome, a key biological source of fecal VOCs. Previous studies have shown that captivity alone impacts the fecal microbiota of cervids (28, 30), and that CWD infection can further alter the microbiome (28, 31). Another possible explanation lies in the dietary habits of infected cervids. Research shows that cervids with late-stage infection struggle to maintain efficient foraging activity (6, 32). This difficulty foraging may prove far less impactful in captive WTD, provided ample food by farmers, than in wild populations, explaining the greater discrimination observed by fecal volatile analysis.

One limitation of this study is that we did not assess fecal samples for the presence of CWD prions using amplification techniques such as PMCA or RT-QuIC. Given the known temporal variability in prion shedding throughout the disease course (8), it is possible that VOC signatures are influenced not only by disease status, but also by the presence or absence of detectable prions at the time of sampling. Such presence could affect VOC profiles directly, through biochemical interactions, or indirectly via host responses or microbial community changes. In addition, we acknowledge that our study was underpowered, which limits the strength of the conclusions that can be drawn. However, these preliminary findings provide important estimates of variability that will inform sample size calculations and guide the design of future, adequately powered validation studies. Integration of prion detection with VOC profiling in larger, well-characterized cohorts will be a critical next step to advancing this approach toward diagnostic application. Rather than focusing on individual compounds, future work should emphasize pattern recognition and statistical modeling approaches that can accommodate biological and environmental variation.

Detecting early-stage CWD, ideally prior to symptom onset, would be optimal for long-term control of spread. While further targeted investigation is required, the results from the current data set demonstrate that VOCs during early-stage CWD bear similarity to late-stage CWD. This is especially apparent in samples from wild cervids (Fig. S5). These data suggest that fecal VOC analysis is a promising method for early detection of CWD in both captive and wild cervids. Adaptation in methodology is necessary for non-invasive implementation, such as comparison against proximal environmental samples to control for non-fecal VOC contamination. Nevertheless, following further optimization, non-invasive detection of CWD-associated volatiles *in situ* among WTD populations could enhance wildlife disease monitoring efforts. Modern technologies such as electronic nose (e-nose) sensor arrays (25) or miniature mass spectrometers could further enhance the speed and efficiency with which CWD is detected and its spread limited.

## MATERIALS AND METHODS

### Fecal sample collection and preparation

Fecal samples were sourced from an existing repository located within the Wildlife Futures Program at the University of Pennsylvania’s Veterinary School. All samples for this study were collected by either certified CWD sample collectors (trained through the USDA and Department of Agriculture), a veterinary pathologist, or USDA staff.

Captive WTD for this study were naturally infected CWD-positive and CWD-negative animals obtained from planned depopulations in the State of Pennsylvania (Table S2; Fig. 2). Wild WTD samples for this study were collected from Pennsylvania (Table S2; Fig. 2) hunter harvests, roadkill, or clinical suspects for CWD.

Animals with samples collected had their feces removed postmortem to ensure that samples were associated with that individual animal. No animals were used for this study that were not sampled/collected within 24 h of death and if body condition was intact and without obvious signs of bloating and decay. Samples were not collected when outside temperatures were above 80°F during the day. Feces were collected manually from the rectum of each animal using a new nitrile glove to prevent cross-contamination. Feces were then placed into sterile whirl-pak bags and stored at −80°C until aliquots were removed for this study.

The CWD status of the samples was determined as follows:

- Wild deer (roadkill and hunter harvest)—samples were tested at the Pennsylvania Animal Diagnostic Lab (PADLS) New Bolton Center, University of Pennsylvania, School of Veterinary Medicine by proficiency-tested technicians. RPLNs were screened by ELISA (12). If samples were positive on initial screening, testing was repeated an additional two times by ELISA. If all three tests were positive, IHC was performed on the RPLN for confirmation of abnormal protein deposition by a board-certified pathologist.
- Wild deer (clinical suspects)—samples were tested at the PADLS New Bolton Center lab. RPLNs and brainstem (obex) were tested immediately by IHC for confirmation and read by a board-certified pathologist.
- Captive deer—due to the higher likelihood of CWD detection in captive deer, samples bypass ELISA screening and both RPLN and brainstem (obex) were tested by IHC at the USDA National Veterinary Services Laboratory in Ames, IA, as previously described (33). CWD-positive samples were further subdivided into those with disease-associated prion protein detected in both obex and lymph nodes and those with disease-associated prions in lymph nodes but not in obex (LN), which were considered as possible cases of late and early stages of the disease, respectively. These distinctions were included in some of the analyzes as indicated in the text and figures (Fig. S2 and S5; Table S2).

From each sample, after a partial thaw, a 300 mg aliquot was placed into a 20 mL glass vial (Supelco, Sigma Aldridge, Poole, UK), sealed with magnetic screw caps (8 mm center hole with silicone/polytetrafluoroethylene septum), and samples were frozen at −80°C until analyzed.

### Fecal headspace analysis

The method for semi-quantitative analysis of volatile compounds in fecal samples was performed using headspace solid-phase microextraction GCxGC-MS (HS-SPME-GCxGC-MS). HS-SPME extractions and injections were carried out using a PAL RSI autosampler (SepSolve Analytical, Peterborough, UK).

SPME fiber coated with carbon-WR/PDMS (Supelco, Bellefonte, PA, USA) was used. Before use, fiber was conditioned as suggested by the manufacturer. A SPME fiber blank was performed at the start of the sampling batch during the sequence, ensuring the absence of carry-over.

#### Preloading of fibers

Preloading of the SPME fiber with the standard reagent was performed by placing diluted reagent (1,157 µg of 4-fluorobenzaldehyde in 1 mL of water) in a 20 mL sealed headspace vial. The vial was heated at 60°C for 1 min. The SPME fiber was then exposed to the headspace vial for 30 min with agitation at 300 rpm.

#### HS-SPME fecal VOC sampling

The frozen fecal samples were first thawed at room temperature. Samples were equilibrated for 60 min at 60°C with agitation at 300 rpm before 30 min extraction using the preloaded SPME fiber. After absorption, headspace volatiles were transferred to the GC injection port for 2 min. The injector was equipped with an Agilent 0.75 mm i.d. Ultra inert glass liner and operated in splitless mode at 250°C. Desorbed volatile compounds were separated in an Agilent 7890B GC system, fitted with a flow modulator and a three-way splitter plate coupled to a flame ionization detector and a time-of-flight mass spectrometer with electron ionization (SepSolve, UK). Chromatographic analysis was performed using a Stabilwax (30 m × 250 µm ID × 0.25 µm df) as the first dimension (1D)-GC column and an Rtx-200 MS (5 m × 250 µm ID × 0.1 µm df) as second dimension (2D)-GC column, both purchased from Restek (Bellefonte, PA, USA). The following GC oven temperature program was used: initial temperature 40°C was ramped to 260°C at 3°C/min and held for 1 min. The total run time for the analysis was 70 min. Helium carrier gas was flowed at a rate of 1.2 mL/min. The flow modulator (Insight, SepSolve Analytical, UK) had a loop with dimensions 0.53 mm i.d. × 110 mm length (loop volume: 25 µL), and the modulation time was 2 s.

The GCxGC was interfaced with a BenchTOF-select time-of-flight mass spectrometer (SepSolve Analytical, UK). The acquisition speed was 50 Hz and mass range was 35–350 mass-to-charge ratio (*m/z*). The ion source and transfer line were set at 250°C and 270°C, respectively, and filament voltage at 1.7 V. Electron ionization energy was 70 eV. ChromSpace (SepSolve Analytical, UK) was used to synchronize and control the INSIGHT modulator, thermal desorption, GC, and TOF. The maintenance and condition of the instrument were monitored by running external standard mixtures (SPME Performance Test, Restek, USA) during each sample run. After every sample analysis, the fiber was conditioned for 5 min at 260°C to ensure complete desorption of analytes.

### Untargeted GCxGC-MS data analysis

GCxGC-MS derived chromatograms were first aligned to one user-selected reference chromatogram in ChromCompare+ software version 2.1.4 (SepSolve Analytical Ltd, UK) based on the 1D and 2D retention times and the available spectral information. The alignment algorithm was used to overcome retention time drift observed across the data set.

A tile-based approach was applied to the aligned chromatograms to enable the raw data to be imported into the chemometrics platform directly, without the application of any pre-processing methods, such as integration and identification. The tile size was 26 s in 1D and 0.5 s in 2D with 20% and 25% overlap, respectively. The signal for every individual *m/z* channel of each tile was integrated for comparison across every chromatogram in the data set. This process generated a list of features labeled according to the tile retention times and *m/z* channel.

The data matrix was cleaned of possible artifacts and siloxane derived from the sorbent filament and filtered so that individual *m/z* channels with <30,000 intensity were removed. A feature was retained if it was present in more than 50% of the samples in either group (e.g., CWD positive vs CWD negative), and data were then normalized using the pre-loaded internal standard.

Data reduction was performed using the proprietary feature selection algorithm in ChromCompare+ software. The algorithm uses a multivariate method to consider the covariance between features (34); this approach retained the most significant features of the known sample classes. Unique peaks were putatively identified via comparison with the NIST20 library based on the combination of the mass spectra similarity match ≥60% and further identity confirmation was obtained with pure standards.

### Machine learning

Machine learning was applied to the most significant features retained for both wild-type and captive-type samples. Both models utilized a random forest classifier, implementing fivefold cross-validation with an 80/20 train-test split. We used the sklearn.ensemble.RandomForestClassifier to determine feature importance through Gini importance (also known as mean decrease in impurity). Gini importance is a measure that indicates how often a particular feature is used to split the data across all trees in the forest. It provides insights into the most influential features for the classification task by summing the impurity decrease from each feature across all trees. This helps identify the key features that drive the model’s predictions, offering valuable understanding of the underlying data structure and contributing significantly to the model’s performance.

### Statistics

Statistics and all data analysis were performed in GraphPad Prism 10.2.1. Box and Whisker plots display the median and whiskers display minimum and maximum values. Statistical significance of differences in VOC levels between groups was tested by means of unequal variances *t*-test with Welch’s correction. The following symbols are used to denote significance: *P < 0.05, **P < 0.01, ***P < 0.001, and ****P < 0.0001. PCA was used to visualize the variance between samples given our selection of important biomarkers. Eigenvectors and proportion of variance for each PC and Fig. 3 and 4 are presented in Tables S3 and S4. The discriminative power of the VOCs identified during the untargeted search after feature selection was assessed by ROC curve.

## Data Availability

Normalized peak areas of CWD-associated volatiles found in fecal samples from captive and wild white-tail deer can be found in Tables S5 and S6, respectively.

## SUPPLEMENTAL MATERIAL

Fig. S1 to S5 msphere.00351-25-s0001.pdf Supporting PCA plots and ROC curves.

Supplemental tables msphere.00351-25-s0002.xlsx Tables S1 to S6.

ASM does not own the copyrights to Supplemental Material that may be linked to, or accessed through, an article. The authors have granted ASM a non-exclusive, world-wide license to publish the Supplemental Material files. Please contact the corresponding author directly for reuse.
