# Supplementary material for: Sensitive, non-invasive detection of chronic wasting disease in wild and captive white-tailed deer using fecal volatile profiling
Source: mSphere. 2025 Aug 18;10(9):e00351-25. doi: 10.1128/msphere.00351-25 (PMC12482149; doi:10.1128/msphere.00351-25)
Supplement: Fig. S1 to S5 — Supporting PCA plots and ROC curves. [file msphere.00351-25-s0001.pdf]

SUPPLEMENTAL FIGURES

Sensitive, non-invasive detection of chronic wasting disease in wild and captive white-tailed deer using fecal volatile profiling

Amalia Z. Berna[1], Tzvi Y. Pollock[1], Yang Liu[1], Michelle Gibison[2], Amritha Mallikarjun[3], Joey Logan[4], Cynthia M. Otto[3,5], Audrey R. Odom John[1,6*]

[1]Division of Infectious Diseases, Children's Hospital of Philadelphia, Philadelphia, Pennsylvania, USA

[2]School of Veterinary Medicine, Wildlife Futures Program, New Bolton Center, University of Pennsylvania, PA, USA

[3]School of Veterinary Medicine, Penn Vet Working Dog Center, University of Pennsylvania, PA, USA

[4]Department of Biomedical and Health Informatics, Children's Hospital of Philadelphia, Philadelphia, Pennsylvania, USA

[5]School of Veterinary Medicine, Department of Clinical Sciences and Advanced Medicine, University of Pennsylvania, PA, USA

[6]Perelman School of Medicine, University of Pennsylvania, Philadelphia, Pennsylvania, USA

***#Correspondence:**

Audrey R. Odom John MD PhD

Children’s Hospital of Philadelphia

3501 Civic Center Blvd

CTRB 10100

Philadelphia PA 19104-4318

Email: johna3@chop.edu

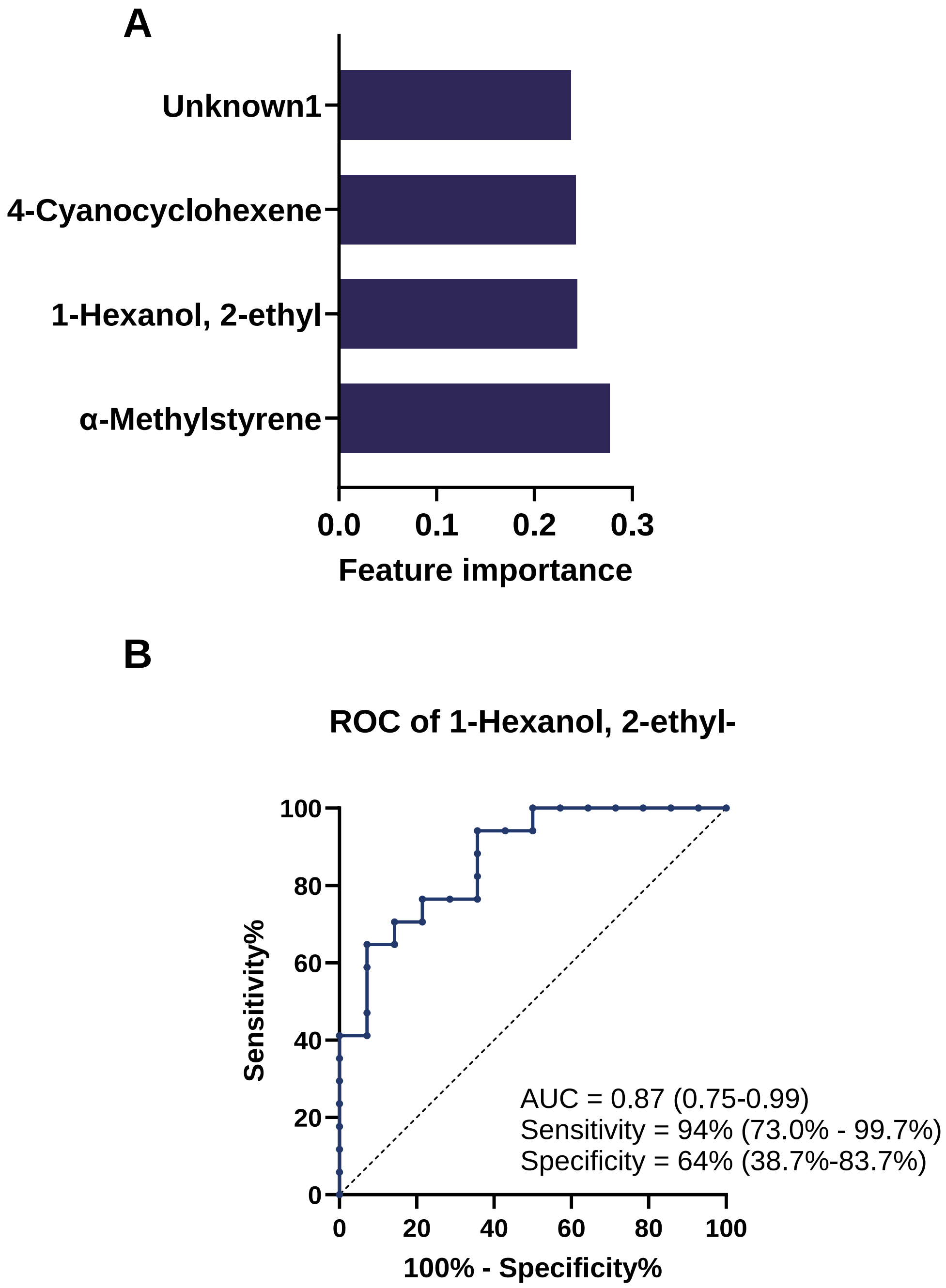

Figure S1. Volatiles discovered in captive WTD.

A.) Feature importance of the four discriminant volatiles was determined using Random Forest Classifier with Gini importance. B.) Receiver operating characteristic (ROC) curve with 95% confidence intervals for AUC, sensitivity, and specificity using 1-Hexanol, 2-ethyl alone to predict infection status.

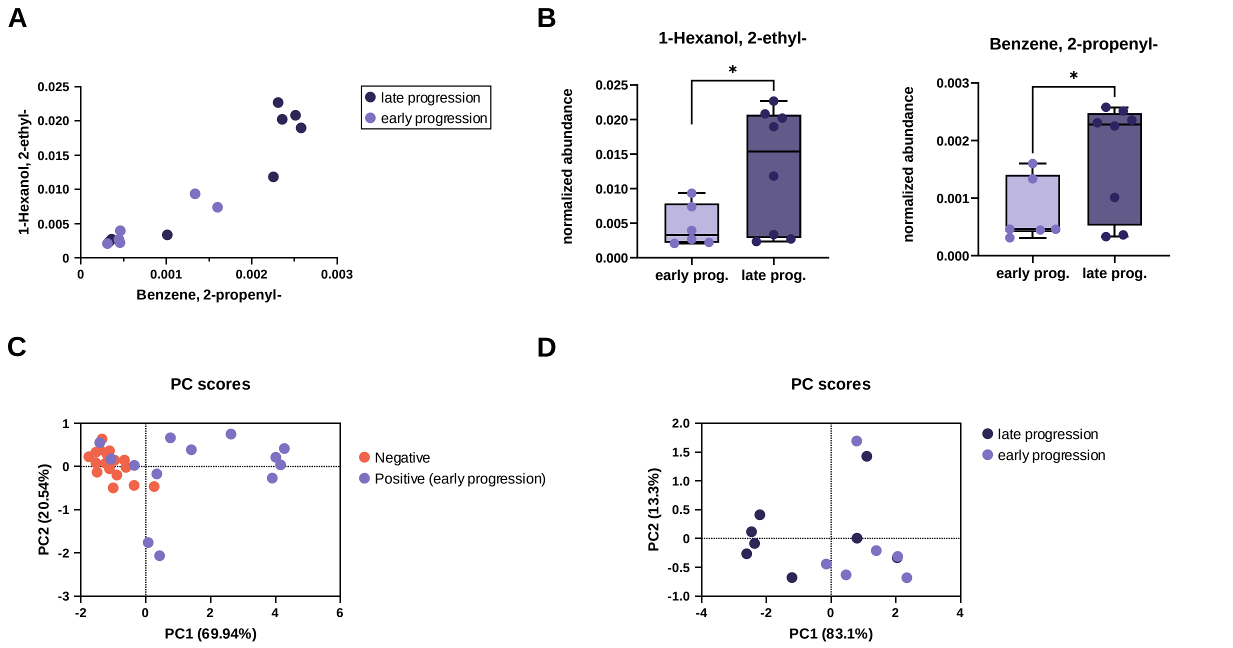

Figure S2. Early and late progression CWD differ in VOC abundance in captive WTD.

A-B.) The relative abundances of both 1-Hexanol, 2-ethyl and Benzene, 2-propenyl during early (N = 6) and late (N = 8) stage CWD infection are compared. Abundances are normalized to internal standard control. C.) Principal components analysis visualizing the distances between early stage positive and negative fecal sample volatile profiles. D) Principal components analysis visualizing the distances between early (N = 6) and late (N = 8) stage CWD fecal sample volatile profiles * $p \leq 0.05$

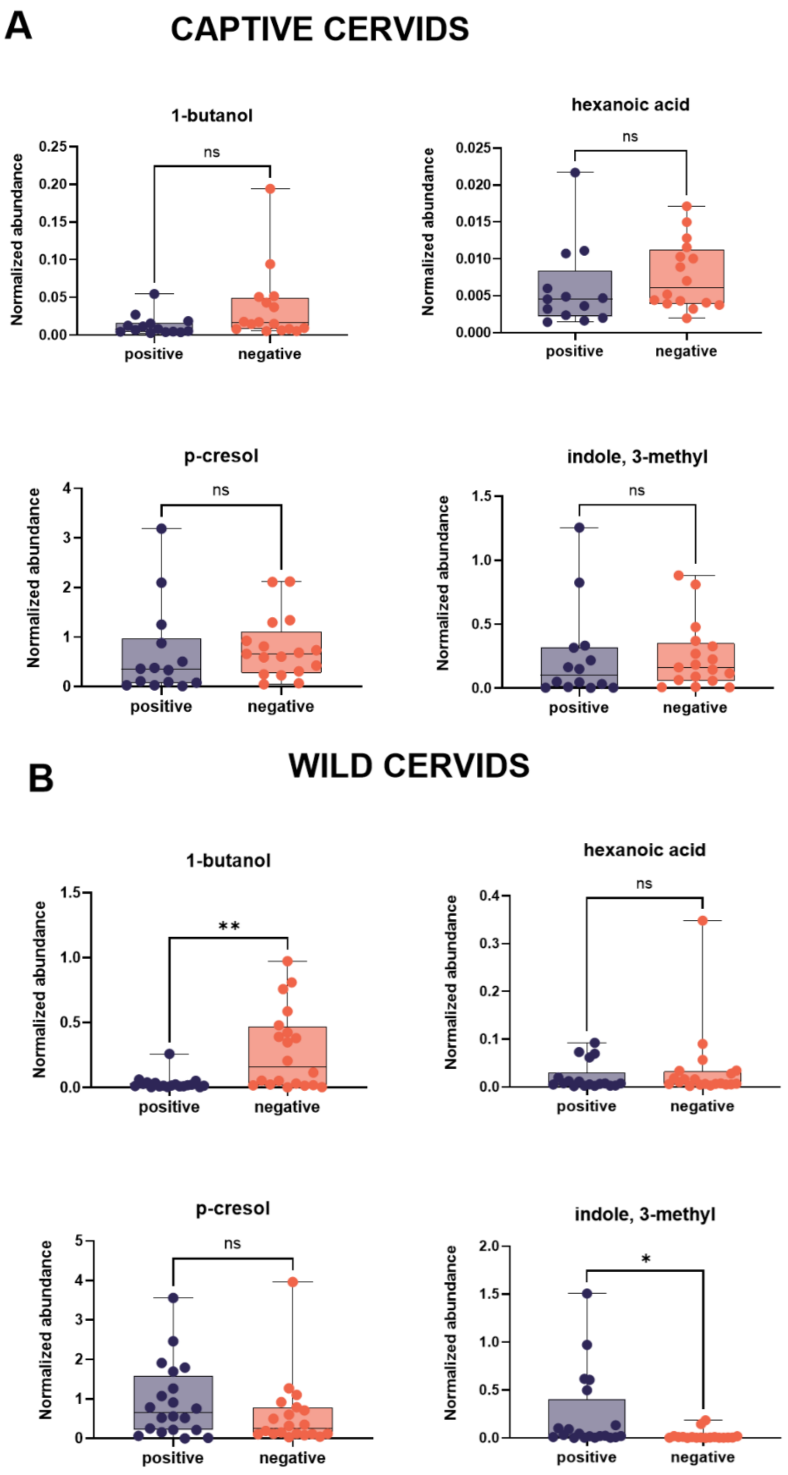

Figure S3. Previously described volatiles do not discriminate between CWD positivity in fecal samples. Abundances of 1-butanol; hexanoic acid; p-cresol; and indole, 3-methyl were compared between positive and negative fecal samples from captive (A) or wild (B) WTD. Abundances were normalized to internal standard control. ns = no significance, * $p \leq 0.05$.

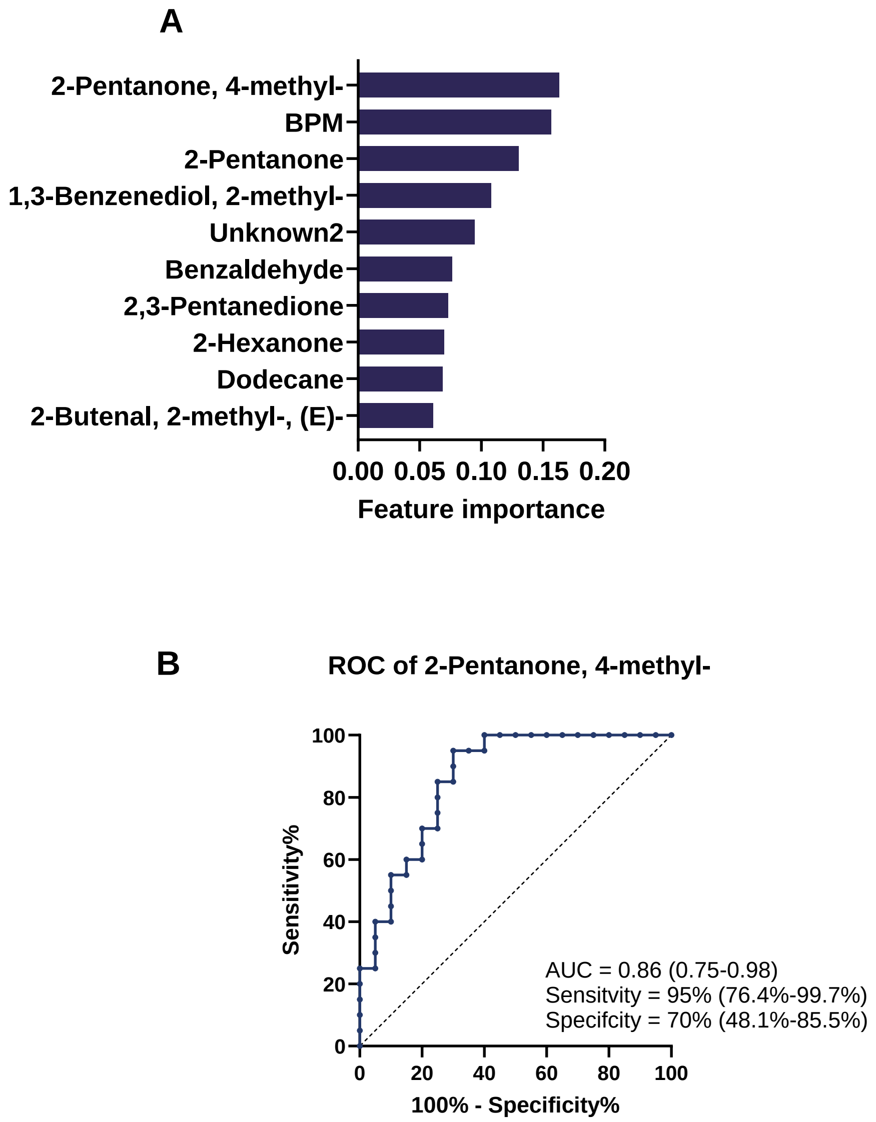

Figure S4. Volatiles discovered in wild WTD. A.) Feature importance of the ten discriminant volatiles was determined using Random Forest Classifier with Gini importance. BPM= Benzyl alcohol, p-hydroxy-α-[(methylamino)methyl] B.) Receiver operating characteristic (ROC) curve with 95% confidence intervals for AUC, sensitivity, and specificity using 2-pentanone, 4-methyl- alone to predict infection status.

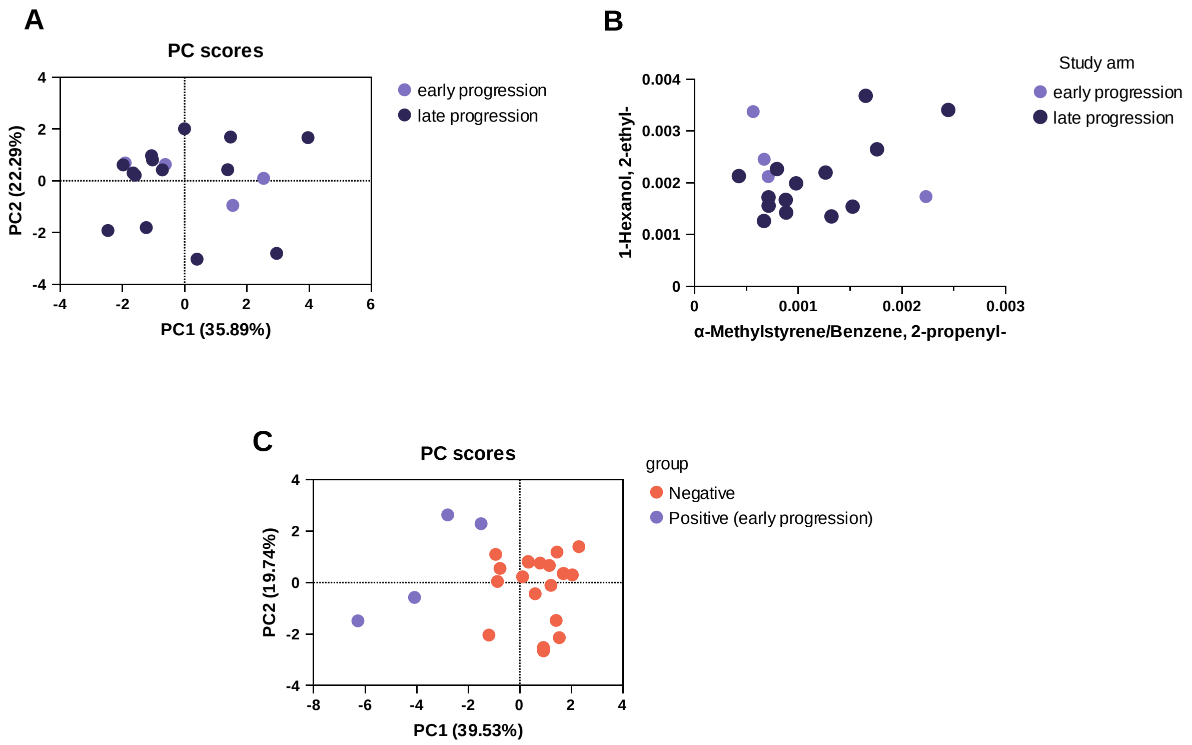

Figure S5. Fecal samples from early progression CWD can be discriminated from CWD-negative samples in wild WTD.

A.) Principal components analysis visualizing the distances between early (N = 4) and late (N = 14) stage CWD fecal sample volatile profiles B.) The relative abundances of both 1-Hexanol, 2-ethyl and Benzene, 2-propenyl during early (N = 4) and late (N = 14) progression CWD infection are compared. Abundances are normalized to internal standard control. C.) Principal components analysis visualizing the distances between early progression positive and negative fecal sample volatile profiles.
